# Lessons learned through respectful maternity care training and its implementation in Ethiopia: an interventional mixed methods study

**DOI:** 10.1186/s12978-020-00953-4

**Published:** 2020-07-02

**Authors:** Anteneh Asefa, Alison Morgan, Meghan A. Bohren, Michelle Kermode

**Affiliations:** 1 School of Public Health, College of Medicine and Health Sciences, Hawassa University, Hawassa, Ethiopia; 2 Nossal Institute for Global Health, School of Population and Global Health, University of Melbourne, Melbourne, Australia; 3 Centre for Health Equity, School of Population and Global Health, University of Melbourne, Carlton, Australia

**Keywords:** Respectful maternity care, Training, Participants, Mistreatment, Childbirth

## Abstract

**Background:**

Improving respectful maternity care (RMC) is a recommended practice during childbirth as a strategy to eliminate the mistreatment of women and improve maternal health. There is limited evidence on the effectiveness of RMC interventions and implementation challenges, especially in low-resource settings. This study describes lessons learned in RMC training and its implementation from the perspectives of service providers’ perceptions and experiences.

**Methods:**

Our mixed methods study employed a pre- and post-intervention quantitative survey of training participants to assess their perceptions of RMC and focus group discussions, two months following the intervention, investigated the experiences of implementing RMC within birthing facilities. The intervention was a three-day RMC training offered to 64 service providers from three hospitals in southern Ethiopia. We performed McNemar’s test to analyse differences in participants’ perceptions of RMC before and after the training. The qualitative data were analysed using hybrid thematic analysis. Integration of the quantitative and qualitative methods was done throughout the design, analysis and reporting of the study.

**Results:**

Mistreatment of women during childbirth was widely reported by participants, including witnessing examinations without privacy (39.1%), and use of physical force (21.9%) within the previous 30 days. Additionally, 29.7% of participants reported they had mistreated a woman. The training improved the participants’ awareness of the rights of women during childbirth and their perceptions and attitudes about RMC were positively influenced. However, participants believed that the RMC training did not address providers’ rights. Structural and systemic issues were the main challenges providers reported when trying to implement RMC in their contexts.

**Conclusion:**

Training alone is insufficient to improve the provision of RMC unless RMC is addressed through a lens of health systems strengthening that addresses the bottlenecks, including the rights of providers of childbirth care.

## Plain English summary

Improving respectful maternity care and eliminating the mistreatment of women during childbirth is a key strategy to improve maternal health. However, there is limited evidence on the effectiveness of respectful maternity care interventions and implementation challenges, especially in low-resource settings. This study examines service providers’ reaction to and experiences of respectful maternity care training and its implementation. Both qualitative and quantitative approaches were used to appreciate how training participants perceived and experienced the training and its implementation in public hospitals. Identification of the challenges service providers experience in implementing respectful maternity care training will help make system-wide and evidence-based preparations, in addition to the training, in order to promote respectful maternity care in health facilities. The training improved the participants’ awareness of the rights of women during childbirth. Participants’ perceptions and attitudes about respectful maternity care were also positively influenced by the training. However, participants believed that the training did not address providers’ rights. Structural issues were the main challenges providers reported when trying to implement respectful maternity care in their contexts. Further health system strengthening actions are required to address structural issues if respectful maternity care is to be improved.

## Introduction

In 2017, almost all (99%) of the 295,000 global maternal deaths occurred in developing regions, 66.3% in sub-Saharan Africa [1]. Evidence shows that improving access to quality and woman-centred care during pregnancy and childbirth substantially reduces preventable maternal and newborn deaths [2, 3]. Respect and dignity, effective communication, and emotional support are key domains of the World Health Organization’s (WHO) vision for quality of care for pregnant women and newborns [4]. These domains are also integral parts of respectful maternity care (RMC) and make a sizable contribution to positive childbirth experience [5]. Furthermore, RMC has been flagged as a potential strategy for reducing preventable maternal mortality and morbidity to accelerate progress towards meeting the SDG targets for improving maternal health [6].

RMC is defined as “the care organized for and provided to all women in a manner that maintains their dignity, privacy and confidentiality, ensures freedom from harm and mistreatment, and enables informed choice and continuous support during labour and childbirth” [7]. Mistreatment during facility-based childbirth may discourage women from giving birth in health facilities [8], and is a violation of their right to health [5]. Although a standardized approach to measuring mistreatment is still evolving, studies from Ethiopia [9–13] and other sub-Saharan Africa countries [14–18] report high levels of mistreatment, including physical abuse. The growing account of the mistreatment of women throughout labour and childbirth globally led the WHO to publish a statement entitled “The Prevention and Elimination of Disrespect and Abuse During Facility-Based Childbirth” [19]. The statement calls for heightened actions and research on RMC and mistreatment to improve women’s access to respectful and quality maternity care services.

Interventions that promote RMC may be multi-dimensional and include components such as RMC training, quality improvement initiatives, maternity open days, community workshops, client service charter, and dispute resolution. In Kenya and Tanzania, a combination of these interventions demonstrated fewer incidents of mistreatment following the interventions [20–22]. However, information on service providers’ experiences of and reactions to RMC interventions, and related factors affecting implementation is limited. Addressing this information gap not only contributes to the promotion of RMC through evidence-based planning but also serves to identify barriers to RMC within the wider health system.

In 2018, an RMC intervention was implemented in three hospitals located in the Southern Nations Nationalities and Peoples Region (SNNPR), Ethiopia as part of a broader study that aimed to identify health system challenges to the implementation of RMC and potential solutions to address these challenges. The broader intervention included: training of service providers, the introduction of wall posters and pamphlets, and post-training facility-based quality improvement sessions. This paper draws lessons from RMC training and its implementation in these three hospitals. We believe that the findings of this study will add to the existing body of evidence that can be used to design and implement RMC initiatives in low-income settings. The effect of the broader intervention on the mistreatment of women during facility-based childbirth is reported elsewhere (Asefa A, Morgan A, Gebremedhin S, Tekle E, Abebe S, Magge H, Kermode M: Mitigating disrespect and abuse during facility-based childbirth: evaluation of respectful maternity care intervention in Ethiopian hospitals, unpublished).

## Materials and methods

### Description of the RMC intervention

The intervention consisted of a three-day off-site training workshop for participants (midwives, integrated emergency surgical officers, nurses, general practitioners, and health officers) recruited from three public hospitals. Development of the training manual happened in three stages: (1) review of the literature on previous RMC training manuals designed for low-income settings [23–25] and preparation of the draft manual by the primary author; (2) review of the draft manual by senior health system and maternal health experts; and (3) final review for content, applicability and contextualization by three local senior maternal health experts. Topics included in the manual are: an overview of maternal health in Ethiopia, human rights and law in the context of reproductive health, RMC rights and standards, professional ethics, and continuous quality improvement. The RMC training used participatory adult learning principles and was delivered through presentations, role play, demonstrations, case studies, individual readings, videos, and a hospital visit. On the last day of the training, a consultative meeting was held with hospital managers, medical directors, and program managers from health departments. The purpose of this meeting was to generate buy-in for the implementation of RMC in the study hospitals. The trainings were held at a University Comprehensive Specialized Teaching Hospital and facilitated by a local multidisciplinary team consisting of the primary author, a senior maternal health expert, and a senior obstetrician-gynaecologist.

### Study design

**Fig. 1:**
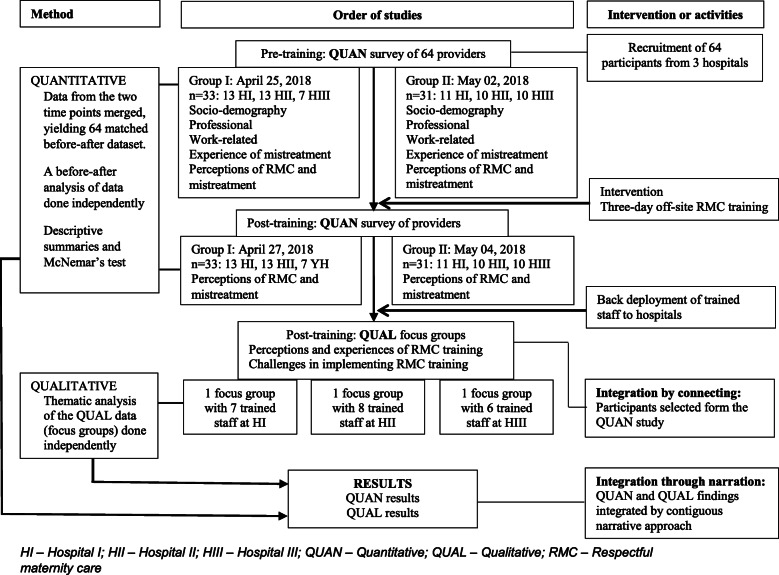
Interventional mixed-methods design

This study used an interventional mixed methods design involving a post-intervention qualitative study (focus groups) which was embedded in a pre- and post-intervention quantitative study (participant survey). Interventional mixed methods supplement an experimental design with a qualitative investigation to: help design intervention procedures, study how participants are experiencing the intervention, and follow up on the outcomes and explain them in more detail [26]. The integration of qualitative and quantitative data can occur before, during, or after the intervention [27]. A pre-intervention survey was conducted first, followed by a similar post-intervention survey with the same participants. Two months after the post-intervention survey, focus group discussions (FGDs) were held with a sub-set of intervention participants (Fig. 1). The quantitative study assessed participants’ experience of mistreatment of women in their facilities and compared participants’ perceptions of RMC before and after the intervention. The qualitative study explored participants’ perceptions of RMC and the challenges encountered when implementing RMC during the 2 months following the training. This article adheres to the guidelines for writing articles of mixed methods recommended by Fetters and colleagues [28]. In this study, the quantitative findings are reported first.

### Quantitative methods

#### Setting

Pre- and post-intervention quantitative surveys were conducted between April and May 2018 immediately before and after the RMC training. The training site is a regional centre of excellence and serves as an in-service training centre for several short-course trainings.

#### Participant recruitment

All health service providers who attend labour and childbirth at the three hospitals were invited to attend the training. All invited service providers from two of the hospitals (26 from Hospital I and 21 from Hospital II) attended the training, in two cohorts to ensure service coverage in the respective institutions. At Hospital III, 5/22 invited service providers did not attend the training due to personal reasons. Service providers participating in the training were invited to take part in the survey. The potential participants were informed about the aim of the survey before the training commenced and were informed that their decision to participate in the survey (or not) would not affect their participation in the training. All (64) service providers agreed to participate (Fig. 1).

#### Instruments and data collection

A self-administered paper-based questionnaire administered in English was used to collect data on the participants’: sociodemographic, professional, and work-related characteristics; observed experiences of mistreatment of women in the 30 days preceding the survey date; and perceptions of RMC and mistreatment. Eight questions representing different categories of mistreatment (non-consented care; lack of information, privacy and confidentiality; physical abuse; verbal abuse; refusal of preference; neglect and discrimination) were used to assess whether participants had witnessed mistreatment of women in their hospital. During the pre-intervention survey, the full version of the questionnaire was used; the post-intervention survey questionnaire only included the section on perceptions of RMC and mistreatment. Following participants’ consent, questionnaires with anonymous codes were put in unsealed envelopes and distributed; participants noted their unique codes, which were subsequently used for the post-intervention survey. The primary author also provided instructions on how to complete the questionnaire page by page. Completed questionnaires were returned in sealed envelopes to maintain anonymity.

#### Data analysis

Survey data were entered into and analysed using Stata (StataCorp, version 15, College Station, TX, USA). Descriptive statistics were computed, and an exact McNemar’s test was performed to analyse pre-post differences in participants’ perceptions of RMC and mistreatment. McNemar’s test is an appropriate statistical procedure for the pretest-post-test analysis of dichotomous variables collected from paired samples; it is used to assess differences on a dichotomous dependent variable between two correlated groups [29].

### Qualitative methods

#### Setting

FGDs were conducted in July 2018 in private meeting rooms at the study hospitals. One of the hospitals (Hospital II) is a primary hospital, whereas the remaining two are general hospitals. All hospitals are comprehensive emergency obstetric care hospitals; based on a review of delivery registers of the hospitals, 12–17% of the total deliveries in 2017 were caesarean deliveries.

#### Sample size and sampling

One FGD was conducted in each of the three hospitals. The criteria for inclusion in the FGD was attending the RMC training. Convenience sampling was used to recruit FGD participants – all training participants who were off-duty and available on the date of the FGD were invited to participate (Fig. 1).

#### Instruments and data collection

A semi-structured interview guide was used during the FGDs, the design of which was based on a review of the literature, the study objectives, and the plan for data integration. The guide was originally prepared in English and translated into Amharic language by the primary author. FGDs were conducted in Amharic by the primary author and were digitally audio-recorded. Participants were provided with compensation for transportation.

#### Data management and analysis

Audio recordings of the FGDs were translated and transcribed from Amharic to English simultaneously by the primary author. The transcripts were imported into NVivo software (QSR International, Version 12 Plus) for management and initial analysis by the primary author. Thematic analysis using hybrid (both deductive and inductive) approach was used and later compared for consistency. The deductive analysis used the semi-structured interview guide as a basis to organize themes and responses whereas the inductive analysis identified themes emerging from the transcripts. The major themes and sub-themes were reviewed vis-à-vis the transcripts and the interview guides by two of the authors, and final themes were agreed upon. The consolidated criteria for reporting qualitative research (COREQ) was used to ensure that important components are reported (Additional file 1) [30].

## Results

### Quantitative findings

Table 1 reports the sociodemographic characteristics of participants. Most of the survey participants were female (68.8%), married (62.5%), 22–29 years old (73.3%), Orthodox Christians (51.5%), and lived in the same town where their hospital was situated (80.0%). Most participants were midwives (79.7%), and 50% had served for more than 5 years as a health professional; 39.1% had served in their current hospital for less than 2 years. On average, 62.5% of participants reported that they worked three or more weekend or night shifts per week.

**Table 1:** Participants’ sociodemographic, professional and work-related characteristics (Survey finding)

Variables		n (%)
Affiliation (hospital)	Hospital I	24 (37.5)
	Hospital II	23 (35.9)
	Hospital III	17 (26.6)
Gender	Female	44 (68.8)
	Male	20 (31.2)
Age (years)	22–29	44 (73.3)
	30–38	16 (26.7)
	Median (IQR)	27 (5)
Place of residence	Same town where hospital is located	53 (82.8)
	Different town	11 (17.2)
Monthly income (in birr)^a^	2700–4200	25 (41.7)
	4201–5500	23 (38.3)
	> 5500	12 (20.0)
	Median (IQR)	4600 (1642.5)
Marital status	Single	24 (37.5)
	Married	40 (62.5)
Religion	Christian Orthodox	33 (51.5)
	Christian Protestant	24 (37.5)
	Muslim	3 (4.7)
	Other	4 (6.3)
Ethnicity	Sidama	139 (70.2)
	Wolayita	17 (8.6)
	Amhara	13 (6.6)
	Oromo	7 (3.5)
	Other	22 (11.1)
Current profession	Midwife	51 (79.7)
	General practitioner	4 (6.3)
	Integrated emergency surgical officer	4 (6.3)
	Nurse	3 (4.7)
	Health officer	2 (3.1)
Service duration as health professional (in years)	< 2	14 (21.9)
	2–5	18 (28.1)
	> 5	32 (50)
Service duration in current hospital (in years)	< 2	25 (39.1)
	2–5	26 (40.6)
	> 5	13 (20.3)
Usual number of night duties per week	≤2	24 (37.5)
	3	23 (35.9)
	≥4	17 (26.6)

^a^*1USD ~ 27 Br (Average between March and April 2018)*

### Providers’ experiences of the mistreatment of women before the training

Participants were asked about their observations of mistreatment of women during childbirth in their facilities within the 30 days preceding the survey date (Table 2). Accordingly, 39.1% of participants reported witnessing fellow health workers conducting vaginal examination for women without maintaining physical privacy. Almost one-third reported witnessing the use of insults, intimidation, threats or coercion directed at a woman or her companions (31.3%). Many observed a healthy newborn kept in a different room from his/her mother (28.1%); and a woman left alone during labour for a long period of time (25.0%). The use of physical force with woman in labour, including forcefully parting a woman’s leg or physically restraining her was also witnessed by 21.9% of participants. More than one-quarter (29.7%) of participants reported that they themselves may have mistreated a woman during childbirth in the previous 30 days; and 29.7% of participants reported that they had felt disrespected or mistreated in their workplace by a patient or other staff member at least once during the same period.

**Table 2:** Participants’ experiences of mistreatment in the past 30 days preceding the training (Survey finding)

Types of mistreatment experienced	Yes, n (%)
Have you seen birth attendants ignore the concerns of a labouring woman?	17 (26.6)
Have you seen a labouring woman left alone for a long period of time?	16 (25.0)
Have you seen a healthy newborn kept in a different room from his/her mother?	18 (28.1)
Have you seen health workers conduct a vaginal examination on a labouring woman without maintaining physical privacy?	25 (39.1)
Have you seen a labouring woman denied foods or fluids when she wanted to have some?	16 (25.0)
Have you heard health workers use insults, intimidation, threats or coercion with a labouring woman or her companions?	20 (31.3)
Have you seen health workers discriminate against a labouring woman based on a specific attribute (age/marital status/ethnicity/education/HIV status)?	14 (21.9)
Have you seen health workers use physical force with a labouring woman (for example slapping, hitting, or tying on a bed)?	14 (21.9)
In your own personal capacity have you done anything that may have disrespected a woman in childbirth?	19 (29.7)
Have you ever felt disrespected or abused in your workplace by a patient or other staff member?	19 (29.7)

### Providers’ perceptions of RMC before and after the training

We assessed the extent to which participants’ perceptions of RMC and mistreatment changed after attending the RMC training. Eight relevant dichotomous dependent variables were collected pre and post the training (paired data) (Table 3). Although not statistically significant, the proportion of participants with positive perceptions of RMC increased after the training in six of the eight domains. Positive perceptions about the belief that it is possible to change how care is structured and provided, and ensuring privacy screens are used did not change (Table 3). The proportion of participants perceiving all eight RMC domains positively before the training was 21.9%, which increased to 35.9% after the training (*p* = 0.08). Beliefs that it is sometimes necessary for service providers to yell at a woman during labour did not show much improvement (21.9% pre-test; 20.3% post-test, *p* = 1.00) (Table 3). The belief that it is not necessary to seek verbal consent from a woman prior to conducting a vaginal examination was 10.9% during post-test (15.6% pre-test, *p* = 0.61). The perception that it is not possible for nurses and doctors to change the way things are done in the labour room got worse (17.2% pre-test; 18.7% post-test; *p* = 0.61).

**Table 3:** Participants’ perceptions of RMC and mistreatment before and after the training (survey finding)

Providers’ perception of RMC and mistreatment	Pre-training	Post-training		***p***-value for Exact McNemar’s test
		Disagree	Agree	
It is not possible for nurses and doctors to change the way things are done in the labour room unless directed by managers	Disagree	47	**6**	1.00
	Agree	**5**	6	
It is sometimes necessary for health service providers to yell at a woman during labour	Disagree	42	**8**	1.00
	Agree	**9**	5	
Ethiopian women understand that health service providers sometimes have to be harsh for the woman’s own good	Disagree	36	**10**	0.54
	Agree	**14**	4	
Husbands should not be allowed in the labour room during the birth of their children	Disagree	34	**11**	0.84
	Agree	**13**	6	
It is sometimes necessary for health service providers to slap a woman during labour	Disagree	54	**3**	0.73
	Agree	**5**	2	
It is not necessary to ask for verbal consent from a labouring woman before conducting a vaginal examination	Disagree	48	**6**	0.61
	Agree	**9**	1	
It is not always possible to screen women to ensure privacy when they are giving birth	Disagree	55	**3**	1.00
	Agree	**3**	3	
Ethiopian women do not want to have a companion of their choice with them when they give birth	Disagree	35	**8**	0.51
	Agree	**12**	9	

### Qualitative findings

Three FGDs were conducted with 6–8 participants per group. Most FGD participants were midwives (81.0%). We identified four major themes in the data analysis: impact of the RMC training; perception of the RMC training; challenges in implementing RMC guidelines; and support required to improve RMC. Corresponding sub-themes that emerged from the second and third major themes are also presented along with illustrative quotations.

### How were providers impacted by the training?

Service providers reported that they had gained new knowledge about the rights of women during childbirth, which included assuming the position of choice, birth companionship, not being yelled at during labour, being provided with information about care, consenting to examination/treatment, and receiving respectful and dignified care.

*Previously, we used to apply force to examine women; sometimes, we also get angry with and shout at them. We have now understood that we must treat women very politely; counsel them on the importance of examination and get their consent before an examination.* [FGD, Hospital III].

Participants discussed that the training influenced their attitudes toward mistreatment. Behaviours that were not perceived as mistreatment before the training were less accepted after the training. This was expressed by the participants in two different ways: as a description of attitudinal change and as self-reported acts of mistreatment. Some of the mistreatment behaviours considered by participants to be “normal” before the training, but not after the training were: denying food during labour, denying birth companionship, denying pain relief measures, not involving women in decision making, and conducting examinations without privacy screens.

*… those things that were considered minor and ignored in routine care, like informing clients about what is being done, are very important. Women should get information about the procedure that they are having, including its advantages and disadvantages. We have now improved our service accordingly.* [FGD, Hospital II].

Participants stated that providers sometimes intimidated or forced women to have a vaginal examination, justifying this because it was considered necessary to avoid negative birth outcomes which could subsequently reflect on their performance evaluations and potentially result in administrative actions. Some women were abandoned because they were perceived to be uncooperative with providers’ requests. This was mentioned as justification for negligence and was used when staff already felt overburdened and burnt out with their job, as a strategy for reducing their workload. Participants mentioned that the training helped them to better understand that these acts were a form of mistreatment, and that they should be avoided.

*… there were clients who refuse an examination and there were some providers who reply ‘if I am not undertaking the examination for you, no one will come and help you’ in response. This is frightening and unprofessional and it is not a usual practice after the training.* [FGD, Hospital III].

Participants described how the RMC training influenced their perceptions of intentional and unintentional actions used while assisting women. Participants previously believed that whatever they did during childbirth was for the benefit of the women.

*There was an attitude that even if I shout at or insult a woman, it is just for her benefit; to encourage her to labour strongly and get the baby out. I used to think I am clean* [correct]*.* [FGD, Hospital I].

The concepts of RMC and mistreatment introduced during the training helped participants to reflect on their own behaviours and take corrective actions where necessary. Participants explained that informal hierarchies between service providers, and between providers and women, were challenged by the training. One participant said that ‘*the provider-patient hierarchy that existed before the training is changed and we [providers] are treating women as our clients, not as patients’* (FGD, Hospital III). Participants also described how they were *‘trying to treat women how they* [providers] *want to be treated’* (FGD, Hospital I). Additionally, participants reiterated that the training helped them to recognize that they must be tolerant when women are perceived to be restless or uncooperative.

*Although a woman speaks something that is very harsh to me, I must be patient, I must swallow* [absorb] *unacceptable behaviours and be tolerant while caring for her, instead of responding to her negatively.* [FGD, Hospital I].

Participants reported that this misconception that women with previous childbirth experiences do not experience labour pain, which can result in poor quality care for multiparous women, was changed by the training. Female participants who had multiple children knew from personal experience that this was a misconception.

*I used to presume that multipara women do not have strong labour pain. I used to get angry at them and say ‘what is wrong with you? This is not your first labour experience’. But, after the training, my attitude has been changed and I am treating multipara women as I treat primiparas; I do not get angry at them.* [FGD, Hospital III].

Majority of participants reported that their motivation for work was positively influenced by the training. One participant mentioned that the feedback she gets from women in response to the good care she provides is motivating for her, ‘i*f you show good behaviour to women, eventually they do not show you a bad one. Thus, I will be very positive and welcoming to them*’.

### How did providers perceive the training?

Three sub-themes were identified during exploration of participants’ perceptions of the RMC training: design and content; training methods; and concern for providers.

#### Design and content

Participants reported that the RMC training manual was well-organized, and the contents were precise and easy to understand. The concepts of RMC and universal rights of childbearing women were reported to have been very new and relevant to the majority of participants.

*I liked the training manual. It is very precise and clear. The training was also delivered in an understandable and clear approach; it was not redundant. In addition, the ideas discussed were what we are working on, practical.* [FGD, Hospital II].

One participant mentioned that it was inappropriate to include issues about sexual abuse in the training manual as such incidents are very rare. Furthermore, some participants were not comfortable with the extent of women’s rights in childbirth, especially the right to refuse procedures. They were concerned that women in the study areas do not have the level of health literacy required to make informed decisions about their care.

*… what was presented as sexual abuse in the case scenarios is a bit annoying. I do not think such events happen in Ethiopia.* [FGD, Hospital III].

#### Training methods

Participants positively endorsed the engaging and participatory approach used by the training, especially the role play, case scenarios, and video shows. Participants valued the professional mix of the training facilitators and appreciated the presence of administrative managers (hospital chief executive officers and medical directors, and program coordinators at zonal levels), which strengthened buy-in to maximize the training’s impact.

*I am happy that senior managers and supervisors were invited to the training. Involving such personnel is a wonderful opportunity to forward our requests and invite their actions.* [FGD, Hospital II].

#### Concern for providers

Participants were concerned that the training did not give adequate attention to the rights of service providers, while it emphasized the rights of women. Accordingly, they suggested that their rights as service providers should also be considered and communicated to service users.

*Providers’ rights should be included, and women’s rights should be revised and context-based. I do not think we can entertain such broad rights of women in our country’s context.* [FGD, Hospital III].

Participants explained that they wanted women and their companions to be made aware of their responsibilities in health facilities when seeking care for childbirth. One participant reported that some clients and companions behave very negatively and abuse providers in a way to claim their rights.

*… where I was working before, there is a community forum and communities were oriented that ‘professionals that dress white gown are meant to serve you [communities]; you can use their service for free’. The people are very innocent; when they come to health facilities and they consider you as their housemaid. Such acts create further friction.* [FGD, Hospital I].

Participants stressed that various training manuals, guidelines, and standard operating protocols, including the current RMC training, predominantly focus on what providers should do for clients. On the other hand, participants reported that there is nothing about what should be done for providers (such as a rise in pay scale, adequate compensation for night shift, and recognition by managers) in response to implementing these multiple instructions.

### Challenges in implementing RMC guidelines

Participants described a range of challenges encountered when implementing the RMC guidelines in practice, including lack of or inadequate infrastructure and supplies, high workload, and women’s and companions’ poor understanding of appropriate behaviour in a hospital setting.

#### Lack of or inadequate infrastructures and supplies

All participants agreed that severe space constraints in the wards made it hard to ensure women’s privacy and allow birth companions. In all three hospitals, multiple women are together in one labour ward (4–6 women) and one delivery ward (3–4 women); all hospitals have only one delivery room. Participants from one hospital mentioned that it is not convenient to walk around the delivery beds if privacy screens are placed in between the beds. Thus, participants believed that it is not feasible to allow a companion for every woman to stay in the wards.

Shortage of supplies like privacy screens, medicines, bed linens, towels, and detergents were the structural drivers preventing the provision of RMC mentioned by participants. Some participants from two of the hospitals described lack of water in the bathrooms as a source of discomfort for women, even if they receive respectful interpersonal care.

Participants from one hospital reported that the hospital does not provide any meal service for women, and as a result, women from rural villages who cannot afford the cost of food for themselves and their companions suffer.

*There was a woman who came having fetal distress and then scheduled for emergency surgery; she stayed here for five days. She did not have money to buy foods. What is the fate of this woman?* [FGD, Hospital II].

#### High workload

Work overload, especially during night shifts, makes it challenging to provide the desired level of respectful care. Night shifts are perceived as long compared to morning and late shifts, and only a small number of providers are available to care for women. Participants said that at times security personnel responsible for controlling overcrowding due to many companions are not available, so nurses and midwives have to assume this role as well.

#### Women’s and companions’ poor understanding of appropriate behaviour in hospital settings

. According to the participants, some women refuse to have procedures like episiotomy and pelvic examination despite having complications such as active bleeding; women who come from rural catchments and lack the knowledge and understanding to make an informed consent. Participants also reported companions’, especially male partners’, lack of consideration for providers to be a problem.

*… there was a nice midwife who was attending a woman. The provider wanted to go to a washroom, but a woman’s companion refused to let him go holding on his neck and saying, ‘you are employed to follow women and you cannot leave my wife for a minute’. This is a huge disrespect of the provider.* [FGD, Hospital I].

### Providers demanded further actions and support to promote RMC

Participants solicited for existent actions in addition to the training to improve RMC in their respective hospitals. These are summarised under three sub-themes: improving infrastructure and supplies; training, capacity building, and motivation; and engaging key stakeholders.

#### Improving infrastructure and supplies

Participants believed that facility managers and zonal and regional health authorities should take proactive action to ensure that all required services and supplies for childbirth are regularly available. Participants emphasized the role hospital managers are supposed to play in this regard, mentioning that the managers should pay close attention to the routine activities of maternity wards rather than only monitoring monthly reports and providing written feedback on these. It was reportedly easier to get a donation from an outside organization than place a supply order via the very long government procurement processes. Participants recommended short-term (partitioning delivery rooms, and renovation) remedies be taken to improve the privacy of women.

*We have informed our managers. The response we get is ‘it is in the process’. You get an item purchased after a long time and the purchased items are very low quality and get dysfunctional in a very short period, even in days.* [FGD, Hospital III].

#### Training, capacity building, and motivation

Participants maintained that training only those in the maternity units is inadequate to improve RMC unless other staff and students in practicum whom women encounter as part of their care, including security officers and cleaners, are trained in RMC. Additionally, it was strongly suggested that nurse, midwife, and medical interns receive a pre-service orientation or training before assuming the responsibility of assisting women at the time of childbirth.

*Respectful maternity care should be everyone’s concern including health professionals, cleaners, security officers, students, and managers. During high caseload periods, there are women who get treated by students only and get discharged. Thus, students should be actively involved.* [FGD, Hospital III].

Participants recommended the recruitment of manpower to balance the existing client load with the number of service providers. Participants also demanded improvement of the pay scale, compensation and benefits, recognition, and visits by managers to be motivated to provide RMC.

*It is after providers get satisfied that they will provide respectful maternity care and make women happier. Thus, we would be grateful if there will be benefit package improvements and adequate motivations by our managers.* [FGD, Hospital III].

#### Engaging key stakeholders

Participants indicated their concern that RMC cannot be achieved by health professionals alone. They said support staff, hospital and higher-level administrators, partner organizations such as teaching hospitals and universities, women, and communities should work together to improve RMC.

*We should be the first actors to improve respectful maternity care. Next, our hierarchical managers and supervisors should ensure the continuity of respectful maternity care service provision. They must come and support us. As said, their support should be in place to make the hospital the best place for women to deliver in receiving respectful maternity care. Everything that needs improvement starting from the gate to the hospital manager should be improved.* [FGD, Hospital I].

## Discussion

This paper presents the analysis of one component (RMC training) of a multi-component intervention (training of service providers, the introduction of wall posters and pamphlets, and post-training facility-based quality improvement). The study complements a growing interest in the promotion of RMC globally and revealed that training of service providers alone is limited in promoting RMC unless it is approached from a health system strengthening perspective. Although the RMC training has positively influenced the perception and understanding of service providers towards RMC, implementations of the RMC recommendations stalled due to diverse barriers. Participants witnessed that the mistreatment of women during childbirth is common in their facilities but cannot be eliminated in their capacities and therefore demanded additional system-wide support by facility managers and beyond.

Participants’ attributed the reasons that women are mistreated during childbirth to one or more of the following domains: lack of knowledge and misunderstanding; normalization of mistreatment; punitive action against uncooperative and emotional women; to gain compliance with required examinations in order to achieve good birth outcomes; and structural issues (inadequate infrastructures and supplies, high workload, and inadequate staff incentive mechanisms). However, the RMC training fell short of addressing the last domain; these structural issues are main drivers of mistreatment and must be intervened to foster the culture of RMC [31].

The ‘health workers for change’ study conducted in four African countries reported that improving knowledge of provider-client relationship was important to instil a positive attitude among providers [32]. That study argued that achieving attitudinal change by trainings alone is futile in the long run, and improving structural issues is also required to achieve sustained change. Another study from Benin reported on reluctance among midwives to institute humanization of childbirth. However, gradual adoption of the new behaviours resulted in increased professional self-esteem and sense of motivation for better care—mainly due to the appreciation from women and family members [33]. In the medium term, we hope that service providers who received the RMC training in the current study may also undergo a similar change process.

Sometimes, the qualitative and quantitative results were incongruent in our study. The survey revealed that participants’ perceptions of individual RMC components did not show significant improvement. However, participants of the FGDs stated that the training positively influenced their perceptions of RMC. This might be due to a social desirability bias because the training facilitator conducted the FGDs; participants might have reported in a way to please the facilitator. Additionally, the survey questions were somewhat limited so could not give a full picture of the changes in perceptions that might have occurred. In contrast, the FGDs allowed participants to describe their perceptions in a more nuanced way. Moreover, the lack of statistical significance in the quantitative assessment might be because providers were perceiving the difficulties they were likely to encounter when trying to improve RMC given their facility’s long-standing structural limitations.

Similar Kenyan and Tanzanian studies found that providers’ ability and willingness to provide RMC was strongly related to how they perceived their work environment including the availability of adequate staff and supplies, career opportunities, support services, and pay [34, 35]. A recent global meta-review indicated that shortage of manpower and lack of drugs and equipment were major bottlenecks to improving the quality of maternal and newborn health care [36]. According to the WHO’s framework for the quality of maternal and newborn health care, using a health system approach to promoting RMC is indicated if real change is to happen because RMC spans all health system building blocks—a deficit in one block eventually affects the remaining blocks thereby subsequently affecting RMC [2].

Birth companionship is an integral part of RMC and a recommended practice throughout labour and childbirth [7]. Birth companions play an important role by providing continuous labour support for women contributing to positive birth outcomes and women’s satisfaction [37, 38]. Participants described that space constraints in the hospitals and birth companions jeopardizing other women’s privacy were deterrents to the inclusion of birth companions in the shared labour wards. Other studies from Ethiopia [9], Kenya [14, 34], Tanzania [21], Guinea [39], and Japan [40] have also reported that the presence of a birth companion is not allowed due to physical structures.

The violation of service providers’ rights reported in this study may not only be a precursor to the mistreatment of women but also demotivating for service providers, which in turn contributes to the provision of disrespectful care [41]. Human rights should apply to both clients and service providers. Therefore, the health system should be organized in a way that enables service providers to enjoy their rights to decent working conditions including adequate wages and staffing, availability of required supplies and equipment, and protection against violent clients [42, 43]. However, participants critiqued the RMC training as lacking a focus on the rights of the service providers. Future RMC initiatives would benefit from inclusive designs that also promote the rights of service providers.

This study benefited from the use of mixed methods design, which helped to identify the range of bottlenecks impeding the implementation of RMC recommendations. Additionally, the use of hybrid technique for the thematic analysis of the FDGs added rigour to the themes identified. We believe that future RMC interventions in similar settings should focus on the identified structural gaps and approach RMC from health system strengthening perspectives to maximize the return of RMC training. However, the study is limited in generating evidence of the challenges service providers might face in implementing RMC recommendations in health centres and tertiary and specialized hospitals as the settings vary in terms of administration and level of service. Additionally, the small sample size, the short implementation period, and the lack of a control group for the quantitative study make attribution of perception changes to the training difficult.

## Conclusions

This study has revealed that RMC training was positively regarded by participants. However, training of service providers alone is limited in promoting RMC because of related system constraints such as trained manpower deployment; essential material and supplies; physical infrastructure (building and space); health professionals’ motivation; and community awareness. Therefore, addressing RMC through a lens of health systems strengthening that promotes a rights-based approach to maternal health services for both women and staff is most likely to successfully mitigate the mistreatment of women during facility-based childbirth.

## Supplementary information

**Additional file 1.** Consolidated criteria for reporting qualitative studies (COREQ): 32-item checklist.

## Data Availability

Reasonable requests can be made to access the data analysed in this study from the corresponding author.

## Abbreviations

COREQ: Consolidated criteria for reporting qualitative research
FGD: Focus group discussions
SNNPR: Southern nations nationalities and peoples region
WHO: World health organization
